# Bipolar disorder and vitamin D deficiency: prevalence, sociodemographic and clinical characteristics

**DOI:** 10.1192/j.eurpsy.2026.10701

**Published:** 2026-07-31

**Authors:** M. Stevanovic, Z. Pavlovic

**Affiliations:** Theraporesistant disorders, Clinic for psychiatry, Belgrade, Serbia

## Abstract

**Introduction:**

In recent years, the possible effects of vitamin D deficiency have been intensely studied in mental health. Studies showed that prevalence of vitamin D deficiency is higher in individuals with psychiatric disorders than in the general population. Vitamin D deficiency has been found to occur frequently in patients with bipolar disorder and has been linked to clinical symptomatology. However, research focusing on the relationship between vitamin D and bipolar disorder is limited, with only a few studies published to date

**Objectives:**

Examination of the prevalence and degree of vitamin D deficiency, as well as the association between sociodemographic and clinical characteristics and vitamin D deficiency in patients with bipolar disorder

**Methods:**

In the retrospective observational study we included 185 inpatients hospitalized at the Clinic for Psychiatry of the University Clinical Center of Serbia during 2022 - 2024 year, with a diagnosis of bipolar disorder, aged 18-65. The data were obtained by analyzing the medical records of patients from the hospital information system “Heliant”. The diagnosis of bipolar disorder is classified according to the International Classification of Diseases - 10th revision. The study was approved by the local ethics committee, based on the Declaration of Helsinki in June 2025.

**Results:**

Hypovitaminosis D was reported in 76.8%; severe deficiency reported 14.6%, deficiency in 36.8% and insufficiency in 25.4% patients. The mean serum vitamin D concentration was 54.79 ± 30.42 nmol/L. Vitamin D deficiency was more frequent during autumn/winter in comparison with spring/summer period (74% vs. 25%). Severe deficiency was observed in patients with lower educational levels.

**Conclusions:**

Our results indicate that many patients with bipolar disorder have vitamin D deficiency. The results of our investigation suggest that any such relationships may be more complex and possibly influenced by a number of confounding factors.

**Disclosure of Interest:**

None Declared

